# When was the last time you cried?

**DOI:** 10.51866/mol.508

**Published:** 2023-11-28

**Authors:** Mohamed-Syarif Mohamed-Yassin

**Affiliations:** 1 MBBS, FRACGP, DipPallMed(Clin.), Department of Primary Care Medicine, Universiti Teknologi MARA, Sungai Buloh Campus, Sungai Buloh, Selangor, Malaysia. Email: syarif8258@uitm.edu.my

**Keywords:** Stroke, Atrial fibrillation, Echolalia, Aphasia, Seizure

*“Anak jantan tak nangis Papa... anak jantan tak nangis"* said Naim to his dad while they were both crying on Naim’s hospital bed, in the final episode of the High Council drama series on one of Malaysia’s paid TV channels. Reflecting on this scene, I honestly cannot remember the last time I cried – perhaps in the early 2000s when my late grandmother passed away. That was before this fatefUl day when my late father, Ayah, as I used to call him, was admitted to a hospital in Seremban. Over 6 days, I cried four times.

Ayah called me around 8 am that morning, telling me that he woke up around 2 am and could not move his left leg. He was able to move all his other limbs, so he went back to sleep. I advised my mother (Mak) to drive him to the nearest hospital in Seremban for further evaluation. I drove down from Gombak to meet them there.

As a doctor (family medicine specialist), I asked Ayah about his symptoms when I met him. He appeared to be his usual self, with no obvious facial or limb abnormalities suggesting that he had a stroke. He said that everything seemed to be back to normal, except for a mild, generalised right leg pain. Upon a quick examination of his nervous system, I confirmed that he had normal muscle strength. His right leg looked normal. He was also able to answer my questions appropriately.

The attending physician, Dr A, confirmed my examination findings with his. However, given Ayah’s known atrial fibrillation, a condition wherein the heart beats irregularly, for which he took a potent blood-thinning medication, an urgent computed tomography (CT) scan of his brain was ordered. At the time, he was 74 years old, and the concern was whether he had a haemorrhagic (bleeding in the brain) or ischaemic (occluded blood vessel in the brain) stroke.

Mak, Ayah and I walked to the radiology department. Ayah then had his brain CT scan done. As a doctor, I felt that this would not show anything significant, just like the countless brain CT scans and reports that I had seen in my career. The report would take about an hour to complete, so we headed to the cafeteria to have lunch.

When I reflected back, Ayah was uncharacteristically quiet during lunch. He cleared his throat several times but did not comment about the food, like he usually does. When I told him I wanted to perform my *Zohor* prayers, he nodded as a signal for me to go ahead. I found this strange, as he would normally join me.

We walked back to the radiology department to collect the CT report. As we walked towards the consultation rooms, I casually reached for the report and read it. I was shocked – there was bleeding in several areas of Ayah’s brain! But how could this be? He was fully conscious and had normal function of his limbs and normal speech. We were in the lift at that time, and for the first time in a very long time, I held Ayah’s hand to support him in case he collapsed.

I started to assess his mental capacities. I asked him the number of medications he normally took – which he knew by heart – but he could not answer. He tried to but nothing came out of his mouth. I asked him, ‘four or five?’, and he repeated ‘four or five’ after me. Oh no, this was echolalia – the repetition of words spoken by others! I then asked him the number of medications he took at night. He counted with his fingers, ‘one, two, three, four’. I was devastated. These were bad signs. Tears started to stream down my cheeks. I quickly walked to a corner of the building, away from Ayah and Mak. Tears continued to stream down uncontrollably. This was my first cry in a long, long time.

With obvious evidence of having just bawled my eyes out, I approached Dr A’s receptionist and told her to communicate the CT findings to him. Dr A told us to head to the emergency department, where he and a neurosurgeon would meet us. I asked for a wheelchair, and we promptly pushed Ayah there. When asked if he was okay, Ayah kept saying that his right leg hurt. He kept slapping that leg, as if trying to ease the pain.

At the emergency department, Ayah was assessed by both specialists and was then admitted to the high dependency unit for further management. He settled in and looked calm. Mak and I continued to ask him questions. He attempted to answer them but could not find the words. This is termed aphasia. He smiled instead. I kissed his hand *(salam)* and left for home with Mak. Little did I know that that would be the last time I saw him conscious.

Soon after reaching my parents’ home in Seremban, I cried for the second time alone in the bathroom. I realised that Ayah’s condition was serious and might get worse.

I then got a call from the consultant stroke specialist, who informed me that Ayah could not move the right side of his body and that his conscious level was reduced. Another brain CT scan was performed, which showed further bleeding spots. He was then transferred to the intensive care unit (ICU).

That night, I was awoken by a phone call from Dr A, asking me to head over to the ICU as soon as I could, as Ayah’s condition was deteriorating. By then, my sister (Kakak) along with my wife and two children had arrived in Seremban. Mak, Kakak and I sped to the ICU. Dr A explained that Ayah had a seizure. He also had a run of an irregular heart rhythm called ventricular fibrillation, which led to a cardiac arrest – his heart stopped beating. The ICU team performed cardiopulmonary resuscitation for about 4 min before his heart rhythm reverted back to atrial fibrillation. He had to have a breathing tube inserted down his trachea (intubation), which was then connected to a machine that breathed for him (ventilator). For this purpose, he had to be sedated. When I saw Ayah unconscious, intubated and ventilated, I broke down for the third time.

Ayah’s condition remained the same even as another brain CT scan showed further bleeding. On day 6 of his illness, the treating team called for a family meeting with Mak, Kakak and me. The team proposed that we try to remove Ayah’s breathing tube and see if he could breathe on his own. If he could not, it would mean that it was his time to go. As I saw Ayah just before he was extubated, I cried again because I knew he would be leaving us very, very soon, and he did, around 12 pm that day. I lost a very important person and a huge influence in my life. I hope I had been a good son, Ayah!

I have not cried since...

